# The enigmatic role(s) of *P2RY8-CRLF2*

**DOI:** 10.18632/oncotarget.22098

**Published:** 2017-10-26

**Authors:** Renate Panzer-Grümayer, Stefan Köhrer, Oskar A. Haas

**Affiliations:** Renate Panzer-Grümayer: Leukemia Biology Group, Children’s Cancer Research Institute, Vienna, Austria

**Keywords:** childhood ALL, *CRLF2*-fusion, *P2RY8-CRLF2*, clonal evolution, *IKZF1* alterations

*CRLF2*, the gene encoding the cytokine receptor-like factor 2, is located in the pseudoautosomal region 1 (PAR1) of both sex chromosomes. Its aberrant expression is the defining feature of an otherwise genetically heterogeneous relapse-prone group of B cell precursor acute lymphoblastic leukemias (BCP ALL) in children and adolescents. The two most common causative genetic defects are, first, an interstitial deletion that juxtaposes the first noncoding exon of *P2RY8* to the entire *CRLF2* coding region and, second, a chromosomal translocation that places *CRLF2* under the control of the *IGH* enhancer [1]. *IGH-CRLF2* fusions occur in a hematopoietic stem/progenitor cell and are considered initiating events while *P2RY8-CRLF2* fusions are caused by illegitimate V(D)J-mediated recombination and therefore only occur in a B precursor cell. Both *CRLF2* fusions often carry additional alterations in *CRLF2/IL7R/JAK-STAT* pathway genes and may cooperatively activate downstream pathways. Interestingly, basically all *IGH-CRLF2* fusions belong to the Ph-like group of B-other ALL while only a smaller proportion of those with a *P2RY8-CRLF2* fusion do [2]. Considering these facts and additional differences in the ethnic background, age, sex, WBC and outcome of patients affected by the one or other fusion, it appears likely that these two fusions define distinct disease entities. They have a particular association with chromosome 21 alterations, either in the form of constitutional trisomy (half of all Down syndrome ALL have such a fusion) or, albeit exclusively for *P2RY8-CRLF2*, in the form of a somatically acquired primary or secondary alteration.

Although *P2RY8-CRLF2* itself is a well-recognized risk indicator for relapse, its specific biological relevance for disease recurrence is still unclear and therefore a matter of ongoing discussion. Outcome data from different study groups are difficult to compare and interpret, because analyzed cohorts, patient numbers, observation periods and treatment regimens differ, as do screening techniques and ascertainment algorithms. To clarify these issues in a systematic way, we evaluated the salient features of *P2RY8-CRLF2*-positive leukemias by quantifying their clone sizes at diagnosis and relapse and correlated them with the respective *CRLF2* expression levels. These analyses revealed that the majority of *P2RY8-CRLF2*-positive clones are small at diagnosis and virtually never evolve into dominant relapse clones. These findings therefore prove that neither the presence of this alteration nor *CRLF2* overexpression alone provide the affected clones with any noteworthy proliferative or selective advantage [3].

**Figure 1:**
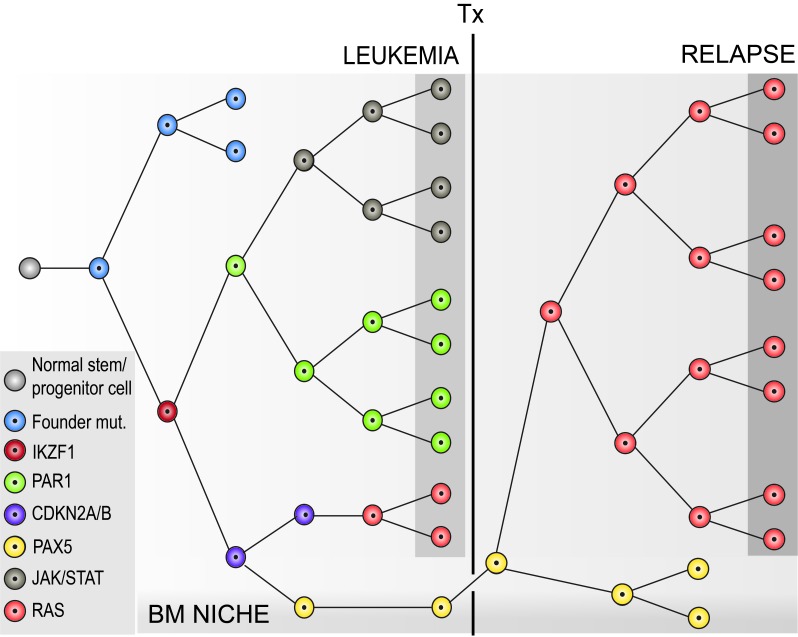
Model for the evolution of *P2RY8*-*CRLF2*-positive leukemia and selection of relapse clones Leukemia-initiating (founder) alterations generally occur in a hematopoietic stem/progenitor cell – and are present in all progenies, while the ensuing RAG-mediated microdeletions evolve only later during early B cell differentiation. Microdeletions affect genes critical for B cell differentiation and tumor suppression. JAK/STAT and/or RTK/Ras pathway activating alterations may emerge during the same time span, are usually subclonal and do not outcompete each other at initial presentation of leukemia. Chemotherapy (Tx) then selects for resistant clones (e.g. *IKZF1* mutant), which are either already present in the dominant clone, as depicted here, or in a small subclone of the initial leukemia. *IKZF1* mutant clones reside in the bone marrow (BM) niche and are there protected from chemotherapy. Only acquisition of proliferation-driving mutations leads to manifestations of the relapse. The model is based on the most striking results of NGS data from our latest study [4]. Each color in the figure represents a particular alteration in a cell and its descendent cells, in which further mutations occur (color code at left bottom of the graph).

As mentioned before, *P2RY8-CRLF2* often evolves as a secondary alteration in leukemias with preexisting, usually primary changes such as a iAMP21, hyperdiploidy and dic(9;20). They concur with other deletions mainly affecting B cell differentiation, cell cycle control and tumor suppression. With the exception of *P2RY8-CRLF2*, these genetic alterations are generally conserved in the dominant relapse clone. To analyze the way in which these clones differ, we studied the mutation and expression patterns at diagnosis and relapse with whole exome and RNA sequencing in 41 relapsing and non-relapsing cases. Although all of them had a predominant *P2RY8-CRLF2*-positive clone at diagnosis, it reappeared in only 13 of 19 (68%) investigated paired cases at relapse. *JAK/STAT* and *RTK/RAS* alterations were present in 78% cases at diagnosis, appeared often sub-clonal, and coexisted together in multiple clones. Such mutations were present in 95% of relapse samples, but only half of the initial mutations were conserved at relapse. Thus, even though both *JAK/STAT* and *RTK/RAS* pathway mutations apparently foster the emergence of disease recurrence in *P2RY8-CRLF2*-positive leukemias, genetic alterations of *IKZF1* are likely to be a more relevant factor in this context, because they already initially prevailed in relapsing cases and increased from 36 to 58% in matched samples. Moreover, these cases also had a typical *IKZF1* transcriptional signature, reflecting a combination of stem cell properties, impaired lymphoid differentiation, enhanced focal adhesion, activation of the hypoxia pathway, deregulation of the cell cycle and drug resistance [4]. These characteristics become also apparent in mouse models in which *Ikzf1-/-* pro/pre-B cells acquire stem cell and adhesion properties including activation of the focal kinase pathway. They further mirror the features in a recent patient-derived xenograft murine model demonstrating the existence of a rare leukemic subpopulation that displays dormancy, stemness and treatment resistance properties as well as persistence in the bone marrow niche [5]. Besides epigenetic changes as likely causes for the plasticity of leukemic cells in generating this small “stem cell” pool, genetic alterations such as *IKZF1* alterations may play a role in fostering such a phenotype (Figure 1).

Based on these insights, we envision *P2RY8-CRLF2* as a “latent or mini driver” mutation [6, 7], a refined term for an earlier described subtype of “passenger” mutation [8]. Such mutations are commonly present in sub-clones, and even though they can positively influence cellular processes, they are not critical for it, but may sometimes substitute for a driver mutation. The success of therapeutically targeting such genes may, consequently, be limited. *IKZF1* alterations, on the other hand, are, especially in this context, attractive actionable targets. IKAROS signaling can be restored by, for instance, casein kinase-2 inhibitors, in case a functional wild-type *IKZF1* allele is still present, or, by inhibition of the activated focal adhesion kinase pathway, a downstream pathway responsible for homing in the niche. Apart from conventional chemotherapy, these approaches could then also be combined with various currently already available JAK/STAT, Ras/MEK/ERK and PI3K/mTOR pathway inhibitors.
